# Barriers to tuberculosis care: a qualitative study among Somali pastoralists in Ethiopia

**DOI:** 10.1186/1756-0500-3-86

**Published:** 2010-03-30

**Authors:** Abdi A Gele, Mette Sagbakken, Fekadu Abebe, Gunnar A Bjune

**Affiliations:** 1 The Department of Social Science, Oslo University College, Oslo, Norway; 2 Section for International Health, Department of General Practice and Community Medicine, University of Oslo, Oslo, Norway

## Abstract

**Background:**

At the dawn of the third millennium, while the control of the second biggest infectious killer in the world (tuberculosis [TB]) is an international priority, millions of pastoralist communities in the Horn of Africa are struggling to access TB care. Prompt diagnosis and treatment of pastoralist TB patients remain to be a challenge in TB control programs in many countries in this region, where pastoralism is a common means of livelihood. Better understanding of community perceptions of TB and its management could help identify reasons for the delay in diagnosis of TB among pastoral communities. The aim of this study is to explore barriers delaying diagnosis among pastoralist TB patients in the Somali Regional State (SRS) of Ethiopia.

**Methods:**

A qualitative study, including 19 respondents was conducted in the SRS of Ethiopia. Participatory Rural Appraisal (PRA) and informal interview techniques were employed to explore pastoralists' migration patterns, their perceptions of TB and their access to TB services. The influence of these factors on the delay of TB patients in receiving biomedical diagnosis was then assessed.

**Results:**

We found that lack of access to formal health services as well as traditional beliefs leading to self treatment were barriers to prompt bio-medical diagnosis of TB among pastoralist TB patients in the SRS of Ethiopia. This study highlights that limited access to TB control programs is the most important barrier in early seeking of biomedical diagnosis of TB among pastoral communities with nomadic pastoralist being the most affected.

**Conclusions:**

Diagnostic and treatment facilities should be established in strategic villages that pastoralist can reach in both dry and wet seasons. Such facilities may alleviate the observed long distance to health facilities and thus long delay in diagnosis of TB. This strategy should be compounded with a community based TB control approach, whereby basic medical training on TB management such as provision of health education, drug distribution and observations is provided to local traditional healers and religious leaders. This approach may improve pastoralists' perceptions of TB, hence eliminating the observed traditional believes associated with TB in pastoralists' context of the SRS.

## Background

The incidence of TB has more than doubled in Africa during the last two decades [1]. This unprecedented increase in TB is attributable to a number of factors, one of the most important being the large number of infectious TB patients who remain undetected and untreated, thereby maintaining the cycle of TB transmission. The ability of TB control programs to contain this growing number of undetected TB patients is constrained by factors that deter TB patients from seeking prompt medical care.

A systematic review of delay in diagnosis and treatment of TB shows that individual health beliefs and level of access to care are some of the factors that influence people's health seeking behavior [2]. Exploring local perceptions and context specific barriers to diagnosis could therefore be of great benefit in the planning and implementation of TB control programs [3].

Ethiopia ranks 8^th ^among the 22 countries with the highest TB burden in the world. Long delay in diagnosis pose a formidable challenge to TB control in Ethiopia [4-8]. These delays have been largely attributed to poor access to health facilities [4-6,8], limited awareness of TB within communities [5,6,8] and health seeking behavior amongst communities that favors the use of traditional healers [4,8] over biomedical approaches.

Pastoralists are migratory people whose livelihood largely depends on livestock with which they migrate seasonally or episodically in search of pastor and water. Two types of pastoralists are widely mentioned in the literature. The first group is nomadic pastoralists who rear livestock; they do not practice agriculture neither do they have any permanent places of abode, but they migrate in a seasonal manner. The second group is agro-pastoralists who engage in unspecialized herding and farming which is mainly a mixed form of subsistence [6,9]. There is an estimated 50 to 100 million pastoralists living in developing countries, 60% of whom are found in Sub-Saharan Africa. Pastoralism is a common source of livelihood in countries in the Horn of African Region, including Somalia, Djibouti, Eritrea, Ethiopia, Sudan and Kenya. In these countries, pastoralists often dwell in border areas; highly volatile and insecure environments that are often beyond the reach of formal health services. Accordingly, disease control activities, including TB control programs, are often absent in these areas [6,10] or are not adapted to the pastoralists' mobile lifestyle [11].

Although many studies on diagnostic delay among TB patients have been conducted in Africa, no attention has been paid to pastoral communities. This study is part of a larger study that investigates socio-cultural attributes in the management and control of TB among pastoral communities in Ethiopia. As part of the study, we quantitatively measured duration of delay in TB diagnosis among pastoralist TB patients [6]. A long delay of TB patients in biomedical diagnosis was documented. This was associated with patient's inadequate knowledge of TB and long distance to health facilities [6]. This part of the study seeks to provide deeper insights into perceived barriers to TB diagnosis among symptomatic TB patients in pastoralist communities within the SRS of Ethiopia. The result of this study adds useful information to our previous study by providing deeper insights into the process leading to the observed long delay in diagnosis of TB among pastoralist patients in the SRS of Ethiopia.

## Methods

### Study area

A qualitative study was carried out in the SRS of Ethiopia between June and September 2007. The SRS is the second largest of the nine regions of Ethiopia, with a land area of 375,000 km^2 ^and an estimated population of 4 million people, 85% of whom are pastoralists. This study identified two groups of pastoralists in the SRS. The first group is agro-pastoralist whose basic livelihood is from livestock, but who also practice non pastoral activities such as farming. The second group is nomadic pastoralists whose economy and livelihood relies exclusively on nomadism and livestock rearing. They do not practice agriculture, nor have they any permanent places of abode.

The SRS of Ethiopia is a site for a long running conflict and insecurity that severely undermine the public sector's ability to deliver basic social services to rural areas. As a result, people in the region are not only exceedingly poor [12] but also bears a disproportionately high burden of TB. In the year 2000, incidence of pulmonary positive TB in the SRS was noted to be 175-250/100000, which is much higher than the national level of 165/100000 [13]. Tuberculosis control program offers short-course chemotherapy which is available free of charge through TB control clinics that are located in major towns. Private sector is rare in the Somali Region. Nonetheless, neither the private sector nor traditional healers are involved in regional TB control [6]. Tuberculosis treatment involves taking a combination of drugs daily under direct supervision by a health worker for 2 months (intensive phase), after which medicines are collected once a month for 6 months (continuation phase). There were 17 diagnostic facilities that were performing acid fast bacilli (AFB) testing throughout the region at the time of the study. These facilities were located in towns and large villages alongside major roads.

### Participants and data collection

During data collection, the first author and a research assistant had two weeks of direct contact with pastoralist patients with the aim of recruiting the most informative individuals for the qualitative part of the study. In the process, we held consultation sessions with pastoralists regarding individuals considered as informative in pastoralists own perspective. Thus, in accordance with purposive sampling techniques, participants were selected due to their knowledge on the phenomenon under study. Determining an adequate sample size in qualitative research is a matter of judgment and experience in evaluating the quality of the information [14]. However, the issues that should be considered are dependent on the heterogeneous or homogeneous nature of the sample population [15]. Pastoralists in the SRS are known to be a closely homogeneous group of people, not only ethnically and culturally, but they also share one language, religion, lifestyle (pastoralism) etc [16,17]. For such a closely homogeneous people, 10 respondents were deemed an adequate sample size to create the intended qualitative product [15]. Thus, 12 pastoralist TB patients were selected to map out the migration routs, while 7 respondents (4 pastoralist TB patients and 3 government officials) attended the interviews; a total of 19 participants. The government officials were included due to their involvement in decision making with regard to health care for the pastoralist communities, thus providing the governmental perspectives on TB service delivery to pastoral communities.

Among the 12 TB patients who participated in the mapping of migration routs, 8 were males and 4 were females. Their ages ranged from 23 to 70 years and they were all Muslim. We employed PRA technique to map out migration routs of pastoralists. The PRA is a qualitative research method increasingly applied in health research. This method has been proven to be highly effective for assessing pastoralists' migration pattern [18]. By using PRA, we aim to provide better insights into the migration pattern of Somali pastoralists and assess their capacity to make use of directly observed treatment (DOT) services. Two groups were formed from the sample: Group 1 consisted of six TB patients from agropastoralists in Qabribayax District, whilst Group 2 comprised of six TB patients from nomadic pastoralist communities inhabiting Dhuxun District. Each group was asked to map out its seasonal migration route. They drew arrows and other features on the sand to illustrate their migration route in both wet and dry seasons. Their original drawings were then transferred to manila paper and finally to microsoft word program.

In order to explore pastoralists' perception on TB, we complemented the PRA with informal interviews. Informal interviews are open interviews where a particular topic is discussed without a predetermined formulation or sequence of questioning, and it permits the collection of in-depth information and exploration of spontaneous remarks by respondents [19]. During the interview, we used interview guide. We also extensively probed participants' responses. Pastoralists were interviewed both in tea shops and under trees based on their preferences. On the other hand, two government officials were interviewed within their own offices while the third one was interviewed within his own home. Each participant was interviewed separately. The group interview was constrained by security concerns in the study area. All interviews were conducted by the first author (AAG) who is a Somali native speaker. Each participant was interviewed twice or more and each interview lasted 2-3 hours. We continued the interview process until it was clear that no new information were emerging from additional interviews, that is, until saturation was reached.

### Analysis

Diary books were used for noting down the interview. The first author and research assistant, both Somali/English speaking, initiated a verbatim transcription immediately after each interview. To validate the content of the interviews, the transcriptions were brought back and verified with the respondents on the following day. The idea was to grasp the real meaning of the concept behind the response and also to ensure that the final transcript represent the original response of study subjects [20].

The transcribed data were read, line by line, by the first author, two senior medical anthropologists and 3 master students who all involved in qualitative health research at the University of Oslo. In the process, data was divided into meaningful analytical units and marked with descriptive words. The coding structure was initiated according to Berkowitz theory [21]. The codes were merged into larger categories and themes. Content from each coded groups were then summarized and illustrated with direct quotes from the interviews. All authors negotiated and agreed the final categories and their content.

### Ethics

Participants signed a witnessed consent for their participation, and the study was ethically approved by Norwegian Ethics Committee and the Ethiopian Science and Technology Committee.

## Results

### Migration route

Study participants mapped out their seasonal migration route. The agro-pastoralists (Group 1), reported that they subsist through a combination of farming and raising livestock. They reported five different seasons each year in their territories, which according to them determine their migration route. These seasons include the following:

#### Jilaal

A dry and very hot season that lasts from January to March. Pasture and water are scarce leading to long migrations.

#### Gu'

The first long rainy season that normally lasts from April to June. Pasture and water are abundant and there is no migration at all. People engage in farming.

#### Xagaa

A dry season that lasts from July to August. It is mildly hot and very windy. Suitable pastures and water are scarce; however mobility is limited and often dependent on the situation.

#### Karan

Short wet season that lasts from August to September. In this period there is no migration at all.

#### Deyr

Second major rainy season that last from October to December. Pasture and water are abundant and farming takes place. There is no migration in this period.

**Figure 1:**
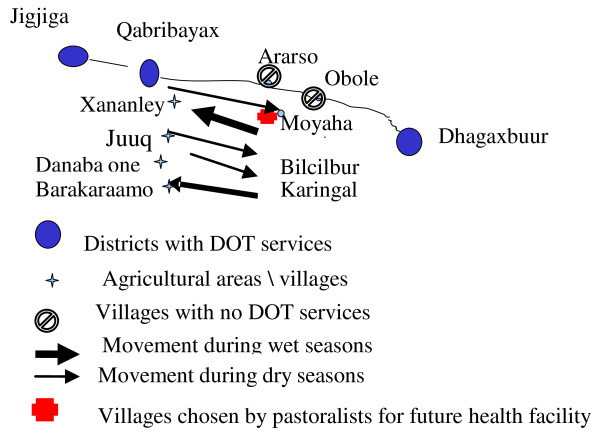
**Seasonal migration of agro-pastoralists in SRS**.

Study participants told that, each year, they receive three rainy seasons that satisfy periodic crop production. During these seasons, agro-pastoralists told that they dwell in permanent villages which are located east of Qabribayax District, namely Xananley, Juuq, Danaba One and Barakaraamo where they engage in small scale farming. These villages are within 20 minutes walking distance of Qabribayax District where there are two health facilities that provide TB care. However, when expected rains fail, and farming becomes impossible, agro-pastoralists migrate eastward into forested areas; namely Karingal, Bilcilbur and Moyaha, in search of pasture and water for their livestock (thin arrows in figure 1, indicates migration during dry season). The length of their stay in those forests varies within each season and it is often determined by the length of the draught. When the rainy season begins, agro-pastoralists migrate back to their permanent villages in Qabribayax (bolded arrows in figure 1, indicates migrations during wet seasons).

Discussions facilitated by the PRA technique revealed that agro-pastoralists reside within walking distance of TB clinics during wet seasons. In this period, they reported that they do not experience major barriers in accessing TB services. During dry seasons however, even though they migrate to a remote area that is 60 km away from health facilities in Qabribayax, they still rely on these facilities when illness strikes.

By contrast, the nomadic pastoralist group reported that their community practices an exclusively nomadic lifestyle. They reported having two dry seasons each year namely *Jilaal*, and *Xagaa*, and two rainy seasons namely *Gu'*, and *Dayr*. They do not experience *Karan*; a brief rainy season that is common in the agro-pastoralists' area of the SRS (semi-highlands).

**Figure 2:**
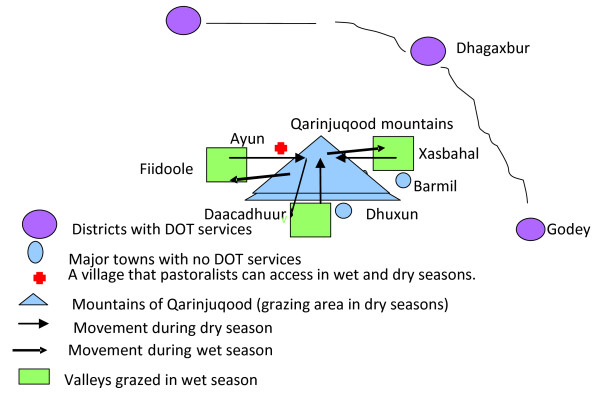
**Seasonal movement of nomadic pastoralists**.

During wet seasons, nomadic pastoralists told that they spread across valleys of Fiidoole, Daacadhuur and Xasbahal, that are located at the base of Qarinjuqood mountain. During these periods, the respondents told that pasture is plentiful everywhere and seasonal swamps and ponds provide water for the livestock. As pasture and water in the valleys dry out, they said that they undertake a long migration, ascending an extended chain of mountains known as the Qarinjuqood mountains (thin arrows in figure 2, indicates dry season migration). According to the respondents, these mountains are not used for grazing in the wet seasons and are therefore rich in pasture during dry seasons. They reported that they stay on top of these mountains for almost six months each year (jilaal and xagaa seasons). Study participants told that because of difficulties in ascending the mountains, months may pass without anybody coming to the area aside from pastoralists searching for pasture and water:

*"The mountains are hard to climb and there are no roads except footpaths. In this period, we don't see anybody except nomads in neighboring hamlets throughout the season" *(Nomadic pastoralist, Jigjiga Health Center).

Once the rainy season begins, pastoralists migrate back to the valleys (wet season migration is shown by bolded arrows in figure 2). The nomadic pastoralists who participated in the PRA were asked to identify the nearest TB facility to their location in both seasons. They identified three DOT facilities, all of which require several days walk to access, regardless of the season.

### Perceptions and management of TB symptoms

Respondents were asked to describe the cause of TB. A majority perceived TB to be the result of internal injury due to hard work or malnutrition. Persistent cough is also perceived to be a normal phenomenon and therefore not necessarily associated with TB. Pronounced weight loss and cough accompanied with blood were symptoms strongly associated with TB. A male pastoralist explains:

*"People cough for years and they still look healthy. If a coughing person becomes rapidly wasted or cough is accompanied with blood, then we suspect the person of having TB" *(Agro-pastoralist patient, Qabribayax DOT center).

When people develop symptoms that are associated with TB, they often seek traditional medicine. The majority of informants reported that they first used traditional herbs, locally believed to cure TB. One respondent who was a traditional healer reported that he treated TB with tetracycline capsules which he believed could cure TB:

*"We have capsule with red and yellow color called tetracycline. We buy it from towns. It must be available in every pastoralist's house because we use it for treatment of both men and livestock diseases such as TB" *(Nomadic pastoralist, Jigjiga DOT center).

If symptoms do not subside after using traditional medicines, patients reported that religious remedies are applied in the form of Koranic verses that are read for the patient. The majority of respondents told that they had tried all available traditional means and they only sought biomedical treatment when they run out of traditional options. One of the participants explains:

"*I tried local herbs, I avoided sex, I tried religious remedies in many occasion and I didn't seek treatment until my situation reached to a point that I couldn't milk camels for my children" *(Nomadic pastoralist, Jigjiga DOT center).

The use of modern medicine is seen as a valid alternative when the available traditional remedies have failed and patients have reached a critical stage of illness. However, even if they seek and partly trust biomedical treatment they still believe that it must be complimented by other practices. Abstaining from sex when TB patients are under treatment were practices reported amongst several respondents. A male respondent explains:

*"My family *[wife] *was moved to Jigjiga area by relatives *[to interrupt sexual contacts] *and my wife will never come here until I finish 8 months TB treatment course"*. (Agro-pastoralist, Qabribayax DOT center).

Some agro-pastoralist patients told that they were planning to stay in the town throughout the 8-month course of treatment, just to abstain from sex. To enforce this practice, nomadic pastoralists apply strict rules. One of the study respondents explained how his nomadic community strictly forbids sexual contact by TB patients;

*"When I go back to my community, elders will assign members of close relatives to scrutinize me such that I never meet my wife privately until I fully recover from TB" *(Nomadic pastoralist, Jigjiga DOT center).

Because TB is characterized by loss of weight, a majority believed that TB could be cured only when treatment is supplemented with the intake of highly nutritious food, a term locally called *baan*. Conventional treatment alone, which is not complemented by nutritious food, is believed to result in a resurgence of the disease.

### Access to health care

According to the respondents, there is a discrepancy in access to health care between the two pastoral groups. When we asked participants about their access to TB services, respondents from the agro-pastoralist group stated that they are semi-urban people and they live in close proximity to health services during wet seasons. However, during dry seasons they migrate to areas far away from health facilities. The majority of the respondents in this group told that in case of illness during dry season they often postpone seeking medical care until next wet season. One respondent told:

*"During dry season, we use traditional medicine. The individuals who are severely sick are sometimes transported by camel to Obole village *[a village along the main road] *where they can hitch for a car to Qabribayax" *(Agro-pastoralist patient, Qabribayax DOT center).

By contrast, nomadic pastoralists reported that they do not have easy access to health care at any time of the year. Despite this, they told that people with symptoms suspected to be TB often seeks medical care during wet seasons. In this period, the increased availability of pasture and water reduces the workload of the herders, providing TB suspects a suitable time to seek treatment. One of the respondents explained:

*"We seek health care during wet seasons because there is not much to do in this period. Livestock can get pasture around the hamlets and water is everywhere. During dry seasons however, families can hardly cope with the dry season burden in our absence" *(Nomadic pastoralist, Jigjiga DOT Center).

A majority of the study participants said that there are no road connections between their dwellings and the major towns where TB clinics are found. Because they occupy a very remote area with no infrastructure, they told that they trek over a hundred kilometer in search of TB treatment. One of the informants explained that their choice of where to seek TB treatment is often influenced by two factors; the presence of relatives to help them whilst they undergo TB treatment and a good price for their livestock. Based on these factors, nomadic pastoralists often seek health care in Jigjiga town (the capital of SRS).

*"The major towns where TB care is found such as Jigjiga, Dhagaxbur, and Godey have similar distance to our area. The difference of Jigjiga is that majority of our people have relatives in Jigjiga and livestock prices are higher" *(Nomadic pastoralist, Jigjiga DOT center).

The majority of patients said that they had to sell livestock to cover their health care costs and living expenses whilst undergoing the intensive phase of TB treatment. Several patients told that they drove heads of livestock all the way to Jigjiga, which stretched the duration of the trip. The majority of study participants reported that it took them between 25 to 28 days from their residence to Jigjiga DOT center. One respondent reported that the nature of the journey excluded vulnerable groups such as women, children and elderly people from accessing biomedical diagnosis and treatment of TB:

*"I am a man and I had a hard time to reach here. It is difficult to bring children and women along. When they *[children and women] *get TB, we treat them with traditional medicine. Sometimes they are cured or they may live with the disease for a long period, or in some cases they die. That is all we can do (...)"*

(Nomadic pastoralist, Jigjiga DOT center).

Although the distance to treatment delivery points was a burden, the main concern of participants was the economic cost incurred by pastoralist families seeking TB treatment. Patients told that they sold an average of 4 camels or 26 goats to cover their daily costs during the intensive phase of treatment. As the livestock is the sole means of their survival, pastoralist had to weigh expenses on their health care against other family needs. One of study participants explained:

*"Some people in our community possess few goats. Such people can hardly seek TB care because they can not pay the cost" *(Nomadic pastoralist, Jigjiga DOT Center).

### Health planners' views regarding provision of TB care to pastoralists

The health officials who participated in this study agreed that pastoralists in SRS can hardly ever benefit from the existing health services. One of the regional health officers explained the challenge that pastoralists pose to current regional health delivery systems:

"Establishment of a health facility in a particular area is determined by population density of that area. For instance, health post which is the lowest in the hierarchy, as a rule serves 3000-5000 people. As pastoralists are sparsely dispersed into large geographical area, they can hardly meet this condition"

Another health officer attributed the absence of TB services in pastoralist dominated areas of the region to their migratory lifestyle:

*"If DOTS facilities are established in pastoralist areas, next day you may not get a single individual, they migrate"*.

The longstanding conflict in the Somali Region and the subsequent high staff turn-over, particularly in the rural areas, was reported by one health official as the reason of pastoralist's poor access to TB care:

*"The health facilities in the rural areas are empty of staff because those areas are hard to reach due to insecurity combined with poor infrastructure. Accordingly, the health workers in the rural parts of the region may not receive salary, sometimes for several months. They often come back to Jigjiga *[the capital town] *and they never go back again"*.

## Discussions

This study identifies barriers and challenges to early diagnosis of TB among pastoralist TB patients in the Somali Region of Ethiopia, and offers examples of measures that may overcome these barriers.

The migration pattern of pastoralists varies from a stable migration where people frequently migrate between two well defined grazing areas to unpredictable migration guided by the availability of water and pasture [9,10,18,22]. Our study shows that seasonal migrations restrict Somali pastoralists' access to health care and thus serves as an important contributing factor to delay in diagnosis of TB. However, the study illustrates that the migration route of Somali pastoralists in the SRS is predictable due to the fact that they migrate between two well defined grazing areas. The forthcoming message from our result is that the pastoralists in the SRS are compatible with conventional health service delivery systems, and they can benefit from sedentary TB clinics that are established in strategic places. This is inconsistent with earlier findings from Kenya where pastoralists were found to migrate wherever there is a permanent source of water during dry seasons [18]. However, it is in line with the finding by Omar [22] who suggested that pastoralists' migration are predictable thus requiring appropriate health care to be planned accordingly. Unfortunately, TB control among pastoral communities is unrecognised priority in the African Horn [23]. This is happening at a period of time when pastoralism remains the sole means of survival for a significant proportion of the populations in the region. The structure of the regional TB programs need to be addressed from an equity perspective to ensure that people living in unique and difficult settings, such as Somali pastoralists, have access to TB care.

Our findings show that participants attribute the etiology of TB to hard work and malnutrition and therefore tend initially to seek treatment through traditional medicine. Beliefs concerning the cause of the disease are a crucial determinant of subsequent health seeking behavior [24]. A study from Sudan shows that lack of awareness of the fact that TB is caused by an airborne, infectious agent increases domestic transmissions and delay TB patients from seeking biomedical diagnosis [25]. The most worrisome finding in our study is the fact that Somali pastoralists consider persistent cough a normal phenomenon, not as a potential symptom of TB. Tuberculosis is only considered when persistent cough is accompanied with blood and severe weight loss. A similar finding has been reported from Addis Ababa, Ethiopia [26]. This reflects a lack of awareness of the contagious nature of the disease, which is a serious public health concern that warrants an urgent intervention through enhanced health education.

Prohibitively long distances to TB services were reported by study participants. Long distance to TB services has earlier been associated with diagnostic delay in Ethiopia [5,6]. Despite the universal access to TB treatments in parts of the world, large number of TB patients, particularly those who belong to the poorest segment of the society, still have limited access to appropriate treatment [26,27]. Today, in the SRS, TB services still reach only a proportion of the regional population. Eighty seven percent of pastoralist TB patients in the SRS were reported to have sought traditional health care for their illness prior to diagnosis [6]. This is largely due to the limitations imposed by insecurity that has been prevailing in the SRS for the last three decades, resulting inadequate health infrastructures in the region, insufficient level of decentralization and shortage of locally available human resources. Community participation in TB control, as part of regional TB program activities has the potential to overcome at least some of these limitations.

Due to the long distance to TB clinics, pastoralist TB patients in our study sacrificed a large number of livestock to meet the high financial expense of seeking TB care. Such heavy financial burdens may discourage TB patients from seeking biomedical diagnosis, and instead encourage the use of traditional methods. Somali pastoralists in Kenya were reported to prefer traditional health care over formal health services. This was because traditional healers were easily accessible for them whereas modern health facilities were not only hardly accessible due to long distance but also lacked the necessary services [9,18]. This is true in pastoralist context of the SRS where pastoralist TB suspects requiring diagnostic testing had to travel over 100 kilometers to nearest DOT facility, while traditional health providers are within close proximity to the pastoralists. In Blantyre, Malawi, 37% of smear positive TB patients consulted a traditional healer prior to diagnosis [28], which has led to some attempts to involve traditional healers in the diagnostic process [29]. To achieve equity and to reach the Millennium Development Goals, regional TB control programs need to identify disadvantaged groups in their society, establish priorities for action based on needs, and to employ targeted interventions to improve access to TB care for pastoral communities.

### Validity of the result

This study reflects the perceptions of limited number of patients who participated in the study and not necessarily those of the whole pastoralists. The failure to generalize the findings of this study to the pastoralist populations is a recognized limitation of the qualitative methods [30]. We interviewed health planners and TB patients but not health providers working in the DOTS facilities. Thus we may miss important information regarding barriers to TB care. The strength of our study is that discussions and interviews took place in a very open atmosphere and answers were not imposed through predetermined questions. Most of the views and opinions were repeatedly expressed among different individuals. Further, this qualitative finding is consistent with the finding in our earlier quantitative study [6], which increases our confidence in the validity of the findings.

## Conclusions

Factors related to socio-cultural perceptions of TB and pastoralists' limited access to health care are the key factors leading to an apparent delay in diagnosis of pastoralist TB patients in Ethiopia. In the SRS where shortage of trained personnel and insecurity are rampant, community-based TB care is an effective and viable option of TB service delivery. Community-based TB care delivery has been found to be cost-effective, and it is a low cost measure that can easily be adapted to diverse areas of need [31]. Accordingly, basic medical training should be given to influential members from pastoralist communities, such as religious leaders and traditional healers. This training should focus on detection of TB suspects, referral systems, drug distribution and observation and dissemination of health information such as causes of TB, its symptoms, how to treat it correctly and where to get this treatment. Originating from, and living among the people, traditional healers and religious leaders will have cultural affinity and accountability. Participation of traditional healers in TB control has been widely advocated [32,33], and significant improvement was reported from Hlabisa (South Africa) where traditional healers were integrated into TB control programs [34]. The integration of traditional health providers to regional TB control programs may encourage early care seeking and adherence to treatment leading to reduction of disease transmission in the Somali Region.

Diagnostic centres should be established in strategic places that people can access the whole year regardless of their migration route. Establishing such centres should be determined through consultation with communities themselves. On the supply side, regular supervision and adequate drug supply should be granted by the Regional Health Authority in collaboration with the NGOs that operate in the pastoral areas.

## Competing interests

The authors declare that they have no competing interests.

## Authors' contributions

AG: Wrote the research protocol, initiated the field work, did the data analysis and drafted the manuscript. MS: Was involved in the write up of the proposal, in the data analysis and the write up and critical reviewing of the manuscript. FA: Was involved in the write up of the proposal and the write up of the manuscript. GB: Was involved in the write up of the proposal and the write up of the manuscript. All authors read and agreed the final draft of the manuscript.
